# Tyrosinase expression in the peripheral blood of stage III melanoma patients is associated with a poor prognosis: a clinical follow-up study of 110 patients

**DOI:** 10.1038/sj.bjc.6601197

**Published:** 2003-10-14

**Authors:** S Osella-Abate, P Savoia, P Quaglino, M T Fierro, C Leporati, M Ortoncelli, M G Bernengo

**Affiliations:** 1 Department of Biomedical Sciences and Human Oncology, Section of Clinics and Oncological Dermatology, University of Turin, v. Cherasco 23, 10126 Torino, Italy

**Keywords:** melanoma-prognosis, melanoma-follow-up, stage III, RT–PCR, tyrosinase

## Abstract

The aim of this study is to define the relationship between the tyrosinase expression in the peripheral blood and the clinical course of the disease in stage III disease-free melanoma patients after radical lymph node dissection. RT–PCR techniques were used to identify tyrosinase mRNA in 110 patients; a total of 542 blood samples were investigated. In all, 54 patients (49%) showed at least one positive result; 13 patients (11.8%) showed baseline positive results: six became negative thereafter, whereas seven showed follow-up positive results until disease progression occurred. One or more positive determinations were found during follow-up in 41 patients with negative baseline tyrosinase. No correlation was found between baseline results and the relapse rate or disease-free survival (DFS), whereas a significant correlation was found between positive tyrosinase results and disease recurrence during follow-up. In fact, 72.9% of positive patients relapsed, but only 19.3% of negative cases did so. The median interval between the positive results and the clinical demonstration of the relapse was 1.9 months (range 1–6.6). Disease-free survival multivariate analysis selected, as independent variables, Breslow thickness (*P*=0.05), lymph node involvement according to the AJCC classification (*P*=0.05) and tyrosinase expression (*P*=0.0001). In conclusion, RT–PCR tyrosinase mRNA expression is a reliable and reproducible marker associated with a high risk of melanoma progression and we encourage its clinical use in routine follow-up.

Tyrosinase, the monoxygenase responsible for the first two steps of melanin biosynthesis (Battayani *et al*, 1993), is one of the most specific enzymes involved in melanocytic differentiation. Its role as a molecular marker in melanoma patients was first proposed by Smith *et al* (1991) in the early 1990s; since then, tyrosinase expression has been applied for the detection of malignant melanoma cells in sentinel lymph node biopsies and investigated in peripheral blood, bone marrow and biological fluids (Ghossein *et al*, 1998; Blaheta *et al*, 2001; Hoon *et al*, 2001).

Several studies (Curry *et al*, 1998; Farthman *et al*, 1998; Ghossein *et al*, 1998; Alao *et al*, 1999; Reinhold *et al*, 2001) have suggested a potential clinical use of the RT–PCR analysis of tyrosinase mRNA to detect circulating neoplastic cells in the peripheral blood of melanoma patients. In a previous paper (Osella-Abate *et al*, 2000), we demonstrated that the majority of untreated stage IV melanoma patients were positive, whereas chemotherapy administration was able to reduce tyrosinase rate, suggesting that tyrosinase may provide a measure of therapy response and be a predictor of disease evolution. However, the prognostic role of tyrosinase expression in the peripheral blood of melanoma patients after the excision of primary lesion and/or nodal dissection has not yet been fully clarified. The meta-analysis performed by Tsao *et al* (2001) suggests that, at the present time, RT–PCR for tyrosinase mRNA is of limited value given the unreliability of the procedure and the lack of data on the outcome of stage I–III positive patients. More recently, Mellado *et al* (2002) and Gogas *et al* (2002) showed that the detection of tyrosinase mRNA in disease-free melanoma patients treated with adjuvant interferon (IFN) is associated with an increased risk of relapse and shorter disease-free survival (DFS).

In this study, tyrosinase mRNA expression has been prospectively evaluated, immediately after surgical treatment and during follow-up, in 110 stage III disease-free melanoma patients, with the aim of defining the relationship between the tyrosinase detection and the clinical course of the disease.

## PATIENTS, MATERIALS AND METHODS

### Patient population

A total of 110 patients with stage III melanoma graded according to the new AJCC classification (Balch *et al*, 2000) (five stage IIIA, 44 stage IIIB, 61 stage IIIC) were enrolled into this study after obtaining informed consent. Inclusion criteria were: (1) radical lymph node dissection (elective, curative or selective) within 3–6 weeks; (2) histological evidence of nodal melanoma micro- or macrometastases; (3) no clinical or radiological evidence of disease at study entry. The clinical characteristics of these patients are reported in Table 1

**Table 1:** Clinical characteristics of patients

**Stage**	**Patients**	**Gender**		**Age (years)**		**Samples**	**Site of primary lesion (*n*)**		**Breslow thickness (mm)**
		**M**	**F**	**M**	**F**				
IIIA	5	2	3	43.5	49	32	Upper extremities	(1)	3.72±1.63
				(range 34–53)	(range 34–60)		Lower extremities	(4)	
									
IIIB	44	23	21	56	49	245	Head	(4)	3.17±3.19
				(range 31–74)	(range 27–61)		Upper extremities	(4)	
							Lower extremities	(15)	
							Abdomen	(2)	
							Back	(18)	
							Others*	(1)	
									
IIIC	61	30	31	54.5	54	265	Head	(3)	3.51±3.11
				(range 24–61)	(range 21–82)		Upper extremities	(6)	
							Lower extremities	(24)	
							Abdomen	(6)	
							Back	(20)	
							Others*	(2)	
									
Total	110	55	55	55	51	542	Head	(7)	3.57±3.08
				(range 27–75)	(range 21–82)		Upper extremities	(11)	
							Lower extremities	(43)	
							Abdomen	(8)	
							Back	(38)	
							Others*	(3)	

* Including mucosal melanoma.

.

All the patients were followed up at our institution. The majority (94 patients; 85.4%) underwent adjuvant treatment with biological response modifiers, according to different nonrandomised pilot trials.

The follow-up schedule was based on clinical examinations every 3 months for the first 3 years after lymph node dissection, every 4 months in the fourth and fifth year, and every 6 months thereafter. Baseline staging procedures for all patients included chest X-rays, abdomen ultrasound, brain, lung and abdomen CT scan, and total-body bone scintigraphy. Chest X-rays and abdomen ultrasound were performed every 6 months for the first 3 years and every 12 months thereafter, and brain, lung and abdomen CT scan were performed every year for the first 3 years, and every 2 years thereafter. Follow-up visits were anticipated in the presence of a clinical doubt of recurrence or on the basis of patients' requests. The follow-up schedule was continued according to plan even in the presence of tyrosinase results.

The first tyrosinase RT–PCR on peripheral blood test (baseline determination) was performed within 3–6 weeks after surgical treatment of regional nodal metastases in all patients (baseline condition). During follow-up, tyrosinase samples were taken at each visit every 3 months for the first 3 years and every 4 months thereafter, or until disease progression.

In all, 50 volunteer age- and sex-matched healthy subjects were used as the control group.

### Blood preparation, RNA extraction and RT–PCR

A volume of 15 ml of blood was obtained from both the groups and collected in EDTA(K_3_) tubes. The first few millilitres of each blood sample was discarded to avoid any possible contamination by normal skin melanocytes. Blood samples were processed within 2 h after collection. The RNA extraction, yield and nested RT–PCR procedures have been described elsewhere (Osella-Abate *et al*, 2000) and all standard steps were taken to prevent carry-over contamination. A negative control (RNA from healthy donors blood) and a positive control (RNA from a melanoma cell line) (Osella-Abate *et al*, 2000) were included with each batch of samples to verify a contamination–free environment and a faultless RT–PCR operating system. The integrity of the RNA for each sample was verified by RT–PCR with primers for human glyceraldehyde 3-phosphate dehydrogenase (GAPDH), as described elsewhere (Osella-Abate *et al*, 2000).

### Preclinical sensitivity testing

The positive control was performed using a melanoma cell line derived from a subcutaneous metastasis, established and characterised in our laboratory (Salomone *et al*, 2000). The same cell line was used to evaluate the sensitivity and reproducibility of RT–PCR with spiking experiments. Duplicate mixtures of mononuclear cells prepared from 10 ml of peripheral blood donated by healthy volunteers were spiked with 10^4^, 10^3^, 10^2^, 10, 1, and 0 melanoma cells. RNA was extracted from these samples and the cDNA synthesised and analysed by RT–PCR. Three sets of experiments were performed. The detection limit was 1 melanoma cell in 10 ml of blood (Osella-Abate *et al*, 2000).

### Statistical analysis

Statistical analyses were performed using the BMDP Statistical Software. The values for each variable were given as mean±s.d. Both parametric (*χ*^2^- and *t*-test) and nonparametric (Wilcoxon test and Mann–Whitney test) tests were used to evaluate any differences between the parameters examined; only ‘*P*-values’ of parametric tests were reported, as the results of the two types of tests were similar.

Disease-free survival was calculated as the time from the baseline tyrosinase sample to the date of progression or last check-up for all patients. Life-table estimates of DFS were derived by the Kaplan–Meier method (Kaplan and Meier, 1958) and statistical comparison was performed by the log-rank Mantel test (Mantel, 1966). The relationship between DFS and prognostic factors was determined by the Cox proportional hazard regression model (Cox, 1972), with a stepwise selection of the significant variables. The two assumptions of the Cox proportional hazard model were tested: (1) The multiplicative relationship between the underlying hazard function and the log-linear function of the covariates (the proportionality assumption), and thus the ratio of the hazard functions for any two patients with different sets of covariates, does not depend on time. (2) The effects of covariates upon the hazard function is log-linear. Six variables were included in the multivariate model: tyrosinase expression, extension of lymph node involvement (according to the AJCC classification), Breslow thickness, gender, age and treatment (treated/untreated). Breslow thickness and age were included as continuous variables, whereas the others were categorical. Two different DFS multivariate analyses were performed according to tyrosinase expression, the first including baseline RT–PCR, and the second including, follow-up results. In the second analysis, a patient was considered tyrosinase positive in the presence of at least one positive determination during follow-up.

## RESULTS

### Clinical results

After a median follow-up of 20. 3 months (range 3–48.3), 47 out of 110 stage III patients (42.7%) relapsed. The median DFS was 14.1 months (range 3–48.3).

The majority of relapsed patients developed distant metastases (34 out of 47, 72.3%); 10 had soft tissue and 24 visceral localizations. Only 13 (27.7%) patients developed a loco-regional relapse (Table 2

**Table 2:** Sites of recurrence

	**No of patients**			**IIIB**		**IIIC**	
**Site of metastasis**	**Positive**	**Negative**		**Positive**	**Negative**	**Positive**	**Negative**
Loco-regional metastasis	9	4		2	2	7	2
			No of metastatic sites				
Distant metastasis	26	8	=1	6	3	12	5
			>1	2	0	6	0
							
Total	35	12		10	5	25	7

): four had local recurrence, seven ‘in transit’ metastases and two a regional node relapse. At the time of writing, no clinical evidence of visceral metastases has been found in any of these patients. No clinical benefit in terms of both relapse rate and DFS was observed in patients who underwent adjuvant treatments.

### Tyrosinase results

All the samples obtained from healthy volunteers were negative for tyrosinase mRNA. A total of 542 blood samples from 110 melanoma patients were investigated. A median of five tyrosinase determinations for each patient was carried out (range 2–17) with a median time to monitoring of 12.3 months (range 3–43 months).

In all, 54 patients (49%) showed at least one positive tyrosinase RT–PCR result (75 out of 542 samples, 13.8%). Overall, 35 patients had only one positive determination and 19 had two or more; among this latter group, the positive determinations were consecutive in 10 patients. According to the time occurrence, six patients were positive at baseline conditions and negative thereafter throughout the follow-up period, whereas seven showed baseline and follow-up positive results until disease progression occurred. One or more positive determinations were found during follow-up in the 41 patients who had a negative baseline tyrosinase. On the contrary, all the determinations performed in the other 56 patients were always negative. An increase in the percentage of positive samples was found from stage IIIA (two out of 32 samples; 6.2%) to IIIB (26 out of 245; 10.6%) up to IIIC patients (47 out of 265; 17.7%).

### Baseline tyrosinase results and clinical course

Only 13 patients (11.8%) showed baseline positive results; the majority of them (nine out of 13) were stage IIIC. Seven of these 13 patients developed a relapse; all these patients showed evidence of circulating melanoma cells throughout follow-up until clinical evidence of disease progression, whereas there was no evidence of recurrence in the six patients who had only baseline positive results followed by negative samples.

No statistically significant difference in the relapse rate was found between patients with baseline positive (Seven out of 13; 54%) and negative results (40 out of 97; 41%) (*χ*^2^=0.319, *P*=0.572). The DFS multivariate analysis showed that the independent variables with a statistical prognostic significance were Breslow thickness (*P*=0.0098) and the extension of the lymph node involvement, according to the AJCC classification (*P*=0.0067) (Table 3);

**Table 3:** Multivariate analysis results

**Variable**	**Coefficient**	**Standard error**	**Risk ratio**	***P*-value**
**(A)**				
AJCC	0.8081	0.2983	2.2436	0.0067
Breslow thickness	0.0010	0.0004	1.0010	0.0098
Baseline tyrosinase*	0.6345	0.4192	1.8860	0.1302
				
**(B)**				
AJCC	0.6186	0.3168	1.8563	0.05
Breslow thickness	0.0007	0.0004	1.0007	0.05
Follow-up tyrosinase*	1.4678	0.3662	4.3399	0.0001

* Tyrosinase negative=0, Tyrosinase positive=1.

baseline tyrosinase results did not influence the outcome of the disease (*P*=0.1302).

### Tyrosinase results during follow-up and clinical course

**Figure 1:**
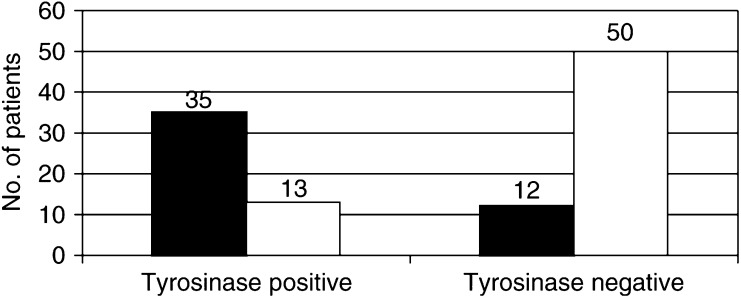
Correlation between tyrosinase results during follow-up and clinical course. A relapse occurred in 35 out of 48 positive (72.9%, 10 stage IIIB, 25 stage IIIC), and in only 12 out of 62 negative patients (19.3%, five stage IIIB, seven stage IIIC) (*χ*^2^=29.566, *P*<0.0001). ▪, PD (progressive disease); □, DF (disease free).

In all, 62 patients had negative tyrosinase results during follow-up, six of whom were positive at baseline conditions and negative thereafter. The remaining 48 patients showed at least one positive determination during follow-up and a statistically significant correlation was found between positive tyrosinase results and disease recurrence. In fact, a relapse occurred in 35 out of 48 positive (72.9%, 10 stage IIIB, 25 stage IIIC), and in only 12 out of 62 negative patients (19.3%, five stage IIIB, Seven stage IIIC) (*χ*^2^=29.566, *P*<0.0001) (Figure 1

). Moreover, nine out of 10 patients with two consecutive positive determinations developed a visceral relapse.

Distant metastases occurred in 26 out of 35 relapsed tyrosinase positive (74.3%) and in eight out of 12 relapsed negative patients (66.7%) (Table 2). No significant difference was found in the tyrosinase expression of treated/untreated patients. The median interval between the finding of positive tyrosinase results and the demonstration of the relapse was 1.9 months (range 1–6.6). At the time of writing, 13 out of 48 positive patients have not relapsed. The positive determination was followed by repeated negative samples in these patients. The median follow-up from the last positive determination is, to date, 13 months (range 3–35).

The sensitivity of the assay (the probability that a positive patient would develop disease recurrence) was 74.5% (95% CI 63.3–83.3%), the specificity (the probability that a negative patient would remain disease-free) was 79.4% (95% CI 71.0–86.0%), the positive predictive value was 72.9% (95% CI 61.9–81.6%) and the negative predictive value was 80.6% (95% CI 72.1–87.4%).

**Figure 2:**
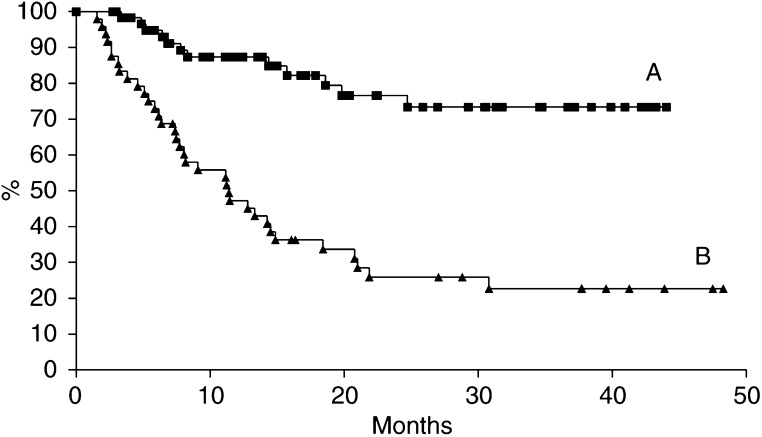
RT–PCR results during follow-up and DFS. Disease free survival was calculated by the Kaplan–Meier method. The statistical significance of the difference in DFS between (**A**) RT-PCR-negative (▪) and (B) RT–PCR-positive (▴) patients was calculated by the log-rank test (*P*<0.001).

The median DFS of follow-up tyrosinase-positive patients was significantly lower than that of negative patients (11.4 months *vs* > 48.3 months, *P*<0.001) (Figure 2

). When patients with only one positive result were compared to those with two, whether consecutive or not, there was no significant difference in DFS. However, the median DFS of positive patients both at baseline and during follow-up was significantly lower when compared to that of positive patients at baseline only or positive during follow-up only (*P*<0.001). Moreover, the median DFS of patients with two consecutive positive determinations was significantly lower than that of patients with two, not consecutive determinations (*P*=0.0188). Disease-free survival FS multivariate analysis was carried out including the RT–PCR results during follow-up instead of baseline. The stepwise model selected as independent variables not only Breslow thickness (*P*=0.05) and extension of lymph node involvement according to the AJCC classification (*P*=0.05) but also the tyrosinase expression (*P*=0.0001) (Table 3).

## DISCUSSION

In this study, the RT–PCR technique was used to detect the presence of tyrosinase mRNA in the peripheral blood of a cohort of 110 stage III disease-free melanoma patients, both at diagnosis and during follow-up, until disease progression. The tyrosinase expression was related with the clinical course, in an attempt to ascertain if the finding of circulating melanoma cells is associated with a higher relapse rate and/or shorter DFS. Our preliminary results (Osella-Abate *et al*, 2000, 2002) suggested a significant relationship between the tyrosinase expression and disease outcome, inasmuch as the progression rate was significantly higher, and the DFS significantly lower in positive patients with respect to the negative ones.

Even if tyrosinase expression in the peripheral blood of melanoma patients has been widely reported in the literature, only a few papers have been specifically addressed to the evaluation of tyrosinase expression both at diagnosis and during the follow-up of stage III disease-free patients. This is of clinical relevance, inasmuch as the majority of disease progressions take place in these patients in visceral sites due to the haematogenous spreading of metastatic cells. Moreover, the monitoring of tyrosinase expression in the peripheral blood could play a relevant role in the evaluation of adjuvant treatments.

There is no agreement in the literature as to the percentage of positive tyrosinase determinations in stage III patients, with a wide range of values up to 60%, according to the different studies (Brossart *et al*, 1993; Kunter *et al*, 1996; Pittman *et al*, 1996; Glaser *et al*, 1997; Farthman *et al*, 1998; Proebstle *et al*, 2000; Reinhold *et al*, 2001). Our results showed a positive tyrosinase expression in 49% of patients. Moreover, the majority of them developed evidence of circulating melanoma cells during follow-up, whereas only 13 (12%) had positive baseline determinations. Mellado *et al* (2002) reported similar results in a cohort of 50 stage III patients treated with adjuvant IFN, showing positive results in 12% before treatment and 46% during follow-up. On the other hand, Gogas *et al* (2002) found a higher percentage of positive results before treatment (50%) in 60 stage IIB–III disease-free patients.

The results of the present study show a striking correlation between the tyrosinase expression during follow-up and the clinical outcome of the disease. In fact, more than 70% of our patients with at least one positive tyrosinase determination during follow-up relapsed, while only less than 20% of patients with constant negative samples did so. The relationship was even more evident when patients with two consecutive positive determinations were considered: all but one (nine out of 10) developed a disease recurrence in visceral sites. Similarly, in agreement with previous data (Osella-Abate *et al*, 2002), tyrosinase expression was found to play an independent prognostic role in influencing DFS, together with the extension of nodal involvement and the Breslow thickness of the primary tumour. The association between tyrosinase results and clinical outcome is also demonstrated by the early detection of disease recurrence after a positive determination. In our experience, this interval is lower (median 1.9 months) than that reported by Gogas (21 months) (Gogas *et al*, 2002), even if, according to the study design, follow-up investigations were anticipated only at the patients' request or due to clinical doubt, and not on the basis of tyrosinase results. The metastatic pattern of relapse, with a higher incidence of visceral localisations in positive than in negative patients, confirms that tyrosinase expression is significantly associated with the haematogenous metastatic pathway. Our data do not provide more insights as to the origin of metastatic spreading in tyrosinase negative patients. Even if it cannot be excluded that the failure to detect circulating melanoma cells may depend on the sensitivity of the technique, the literature data show that tumour cells persist only transiently in the peripheral blood, as demonstrated by sequential analyses taken at a few hour-intervals from the same patients (Reinhold *et al*, 1997). The intermittent shedding of tyrosinase-positive cells in the bloodstream may, therefore, explain the discrepancies observed in patients with visceral relapses.

The intriguing hypothesis that the occurrence of circulating melanoma cells in apparently disease-free patients could constitute the early phase of the metastatic spreading, rather than a consequence of a well-established metastatic localisation, is indirectly confirmed by the finding of tyrosinase-positive results also in a subgroup of patients who have not developed a recurrence to date. Indeed, in our series, 35% of patients showed positive tyrosinase results, preceded or followed-up by one or more negative determinations, and have not developed a clinically objectivable disease recurrence after a median follow-up of more than 1 year.

The biological significance of these findings is still poorly understood. It is not conceivable to associate a metastatic spreading with a positive tyrosinase finding detected more than 1 year before, as all the relapses in positive patients from our series occurred within 6 months from the last positive determination. The conversion from a positive to one or more negative determinations during follow-up could be a consequence of adjuvant treatment, as suggested by Mellado *et al* (2002). Also in our experience, all the positive patients not yet relapsed became negative while under adjuvant treatment. Alternatively, it could be hypothesised that other factors may play a role in inducing and/or inhibiting metastatic growth. Therefore, our previous findings that patients with positive tyrosinase results and high VEGF serum levels show a significantly worse prognosis than those with positive results, but normal VEGF levels, suggest that the modulation of neoangiogenic factors constitutes a crucial key point in early metastatic development (Osella-Abate *et al*, 2002).

In conclusion, the good sensitivity and specificity of the RT–PCR assay, in spite of the possibility that a transient shedding of melanoma cells in the bloodstream could give rise to a false negative determination, confirm that tyrosinase expression is a reliable and easily reproducible marker associated with a higher risk of melanoma progression. We encourage its clinical use in the routine follow-up of melanoma patients. Moreover, we feel that accurate clinical examinations should be carried out together with radiological procedures in the presence of a positive tyrosinase determination, even in the absence of symptoms. Adequate follow-up guidelines for positive patients should be planned, allowing for early observation of any metastasis. From a clinical point of view, we suggest that at least chest X-rays and abdomen ultrasound be carried out and, if negative, that a tyrosinase determination be repeated at a 1-month interval. Brain, lung and abdomen CT, together with bone marrow scintigraphy, should be performed in the presence of a persistent positive determination.
